# Temporal change in plant communities and its relationship to soil salinity and microtopography on the Caspian Sea coast

**DOI:** 10.1038/s41598-022-19863-5

**Published:** 2022-10-27

**Authors:** Galya V. Klink, Ivan N. Semenkov, Yulia D. Nukhimovskaya, Zarema Ul. Gasanova, Nina Yu. Stepanova, Maria V. Konyushkova

**Affiliations:** 1 Institute for Information Transmission Problems (Kharkevich Institute) of the Russian Academy of Sciences, Moscow, 127051 Russia; 2 Lomonosov Moscow State University, Moscow, 119991 Russia; 3 Severtsov Institute of Ecology and Evolution of the Russian Academy of Sciences, Leninskii Pr. 33, Moscow, 119071 Russia; 4 Precaspian Institute of Biological Resources of the Daghestan Federal Research Centre of the Russian Academy of Sciences, Makhachkala, 367000 Russia; 5 Tsytsyn Main Botanical Garden of the Russian Academy of Sciences, Moscow, 127276 Russia

**Keywords:** Biodiversity, Biogeography, Climate-change ecology, Community ecology, Grassland ecology, Restoration ecology

## Abstract

The gradual drying up of saltwater bodies creates habitats that are characterised by changing environmental conditions and might be available only for a subset of plants from the local flora. Using two terrestrial areas with different ages on the Caspian Coast as a chronosequence, we investigated factors including microtopography, ground water level and soil salinity that drive plant community succession after the retreat of the sea. Vegetation of the two key sites appearing after the retreat of the Caspian Sea about 365 and 1412 years ago were compared in terms of both evolutionary and ecological traits of plants. Both edaphic conditions and vegetation differed between the two sites with harsher edaphic conditions and more xerophytes on the elder site. Species that grew only in the ‘early’ site were dispersed across the phylogenetic tree, but their loss on the 'late' site was not random. Species that grew only on the 'late' site were phylogenetically clustered. On the level of microtopography, elevated spots were more densely populated in the ‘early’ site than lowered spots, but on the 'late' site the situation was opposite. The main edaphic factors that drive the difference in vegetation composition between the two sites are likely salinity and moisture. During environmental changes, different plant traits are important to survive and to appear in the community de novo. Microtopography is important for forming plant communities, and its role changes with time.

## Introduction

The decreasing depth of waterbodies is observed mainly in coastal ecosystems of inland depressions located in semi-arid landscapes. In addition to the Aral^1–4^ and Caspian^5–8^ Seas, which are the largest drying waters, this phenomenon is observed in Lop Nur^9–11^, Chad, and smaller territories in Australia^12^, Africa^13^, and the Americas.

Being a closed water body dependent on the inflow of the Volga and the Ural rivers, the Caspian Sea had multiple stages of regressions and transgressions in the past when the sea coast was found at elevations from − 150 to + 50 m above the sea level^14–16^. The latest changes of the Caspian sea level lead to the formation of new dry land on its coasts^17–19^. New territories appear and are inhabited by plants. In the last 3000 years, the northern part of the Caspian Sea was under semiarid conditions^20^. Due to aridity, the salt accumulation was a typical process on the newly formed land. Salt-affected soils with high electrolyte content restrict the development of most plants except halophytes, which can survive. Much is still unknown about plant community succession caused by the reduction in groundwater level resulting from the drying up of the sea. As groundwater level continues to change, such a succession is likely to be highly dependent on changes in abiotic conditions that are not influenced by the community itself. In other words, it might be mostly allogenic. However, some mechanisms of autogenic (i.e. depending on community members) succession may also play a role. For example, large shrubs such as tamarisk might influence the growing conditions in their vicinity^21^, thus influencing the composition of community by facilitation or inhibition mechanisms^22^. Knowledge about the current changes and dynamics of vegetation might help to predict how the territory will look in the future and is critical for developing adaptation strategies to address the challenges posed by climate change and human activities on vulnerable and fragile semi-arid ecosystems^23^.

To understand the processes that drive changes in the structure of plant communities, a complex approach is needed that will consider edaphic factors, as well as ecological traits and the evolutionary history of plants that coexist or change each other during plant community succession^24,25^.

The microtopography with the amplitude of about 30 cm has a strong influence on water relocation and, hence, vegetation in semiarid landscapes of the Northern Caspian Lowland^26–28^. The microhighs receive 4 times less amount of water than microlows, which leads to formation of sparse halophyte vegetation on the tops and dense and diverse vegetation on the bottoms. This phenomenon is well studied at the developed old-aged landscapes at the altitudes of + 0 + 50 m a.s.l. However, the studies on the influence of microtopography on the patchiness of vegetation at the early stages (last centuries) after the retreat of the sea are not well represented in the literature.

In this work, two key sites of different ages were considered as proxy time points of evolution in the area after the Caspian Sea retreated. The two sites—‘early’ (Caspii-2 in our previous works) and ‘late’ (Caspii-1)—were freed from the sea about 293 and 1340 years ago, respectively^29–31^, and are located 1.6 km from each other. The names “early” and “late” reflect that the ‘early’ site is on earlier succession stage than the ‘late’ site. They were compared in terms of vegetation and edaphic factors to study temporal changes of the territory that newly appeared from under the sea in the semi-arid climate. We also compared the vegetation growing on different microtopographic positions (microhighs vs. microlows) both within each key site and between two key sites.

During this work, we tested the hypothesis that on highly saline soils (solonchaks) of the Caspian Lowland, elevations are a harsher environment for plants than depressions in both macro- (i.e. different regressive phases) and microscale (i.e. elements of microtopography—microhighs and microlows).

## Materials and methods

### Key area

The Early and Late key sites were of 30 × 45 and 50 × 50 m size, respectively (Table 1, Fig. S1). Plant communities of the studied key sites were considered as stages of plant colonization of the Caspian Sea coast released from water.

**Table 1:** Characteristics of the studied areas^17,29–33^.

Features	Early key site	Late key site
Coordinates	N 44.5529 E 46.6769	N 44.5412 E 46.6642
Altitude according to the Kronstadt tide gauge, m below sea level	− 25.8	− 24.9 m
Drying age, years BP	365 ± 13	1412 ± 36
Radiocarbon age of the topsoil (0–10 cm) organic carbon, radiocarbon years cal BP (years before present with 1950 as the zero point of the timescale)	293 ± 13	1340 ± 36
Average diameter of the microhighs, m	1–10	1–10
Average height of microhighs, cm	7–16	17–40
Plant community	Tamarisk	Saltwort-suaeda
Total species number	32	24
Topsoil salinity (0–5 cm), dS/m	11.9 ± 8.4 (N = 50)	17.2 ± 14.2 (N = 40)
Subsoil salinity (30–50 cm), dS/m	10.5 ± 1.9 (N = 50)	13.5 ± 1.2 (N = 40)
Groundwater level, m	2.8	> 3.0
Groundwater salinity, g/l	44–48	Not available

As can be judged from the age of soil organic carbon, the short-term flooding in the beginning of the nineteenth century did not impact the ‘late’ key site significantly. Notably, the entire coastal zone of the northern part of the Caspian Sea was repeatedly subjected to flooding associated with storm surges, for example, in 1952 and 1995, the absolute water levels reached about − 24 m for several hours. However, it did not have an influence on the general development of the region’s landscape. At the ‘late’ key site, the lower western and the higher eastern parts were clearly distinguished. The ‘late’ site partially occupied the western slope of the coastal bar formed in the 1880s^34^.

The soils of the ‘late’ site were more saline than soils in the ‘early’ site^29^. At the ‘late’ site, a three-tier plant community consisting of a 0.5–1.0 m high *Suaeda microphylla* in the upper tier, a 0.2–0.5 m high *Kalidium foliatum* in the medium tier, and annual saltworts (*Petrosimonia brachiata, Climacoptera crassa, Suaeda acuminata*) with *Frankenia hirsuta, Psylliostachys spersicicus* and ephemeral plants in the lower herb’s tier occurred^29^. The vegetation of the ‘early’ site was a sparse degrading (deteriorating) community of a 1–1.5 m high tamarisk (*Tamarix octandra* mixed with some *T. laxa*) with annual saltworts (*Petrosimonia brachiata*, *P. oppositifolia*, *Suaeda acuminata*), *Frankenia hirsute*, and *Puccinellia gigantea* in the lower tier with some ephemeral plants^29,30,35^. In the lower tier, two microgroups were clearly distinguished. Open habitats were occupied by sparse stunted halophytic plants (*Petrosimonia, Frankenia, Puccinellia*). Under the crowns of the bushes, dense herb and grass microcommunities grew with a predominance of tall *Puccinellia gigantea* and a few *Limonium caspium*, *L. scoparium*, and *Psylliostachys spicata*, whereas saltworts and *Frankenia* were absent here.

### Field work

In autumn and spring, soils were sampled from 1-m deep boreholes at depths of 0–5, 5–10, 10–20, 20–30, 30–50, 50–70, and 70–100 cm. At the ‘late’ site, 40 auger holes were drilled at a distance of 2 m from each other along a 50-m long transect (26 auger holes) and on different elements of microtopography (14 auger holes)—microhighs and microlows (Fig. S1). At the ‘early’ key site, in total, 58 auger holes were drilled according to a semi-regular grid with an interval of 1 to 5 m from each other.

In autumn, vegetation was described in 2 × 2 m plots throughout the key sites along 50-m long (at the ‘late’ key site, 350 descriptions) and 30-m long transects (at the ‘early’ key site, 345 descriptions) located at a distance of 2–4 m from each other. The descriptions included the number and size of the dominant shrubs, plant species, total projective cover of the herb tier, and the portion of bare ground and plant litter. As the main description of 2 × 2 m plots took place in autumn, only 11 perennial species were found in the ‘early’ site and 15 in the ‘late’ site. In addition, in the spring, the species composition of both sites was studied to compile a list of flora, including not only perennials but also annuals and ephemer(oid)s. The vast majority of species was identified in the field. The formal identification of the plant material used in this study was conducted by Yulia D. Nukhimovskaya and Nina Yu. Stepanova. Voucher specimens were deposited in a publicly available herbarium of the Tsytsyn Main Botanical Garden of the Russian Academy of Sciences (Table S3).

The Latin names of plant species were given according to the S.K. Cherepanov list^36^ based on the Takhtajan system^37^ during the field research and according to open-access continuously updated resources^38,39^ corresponded to the APG IV system^40^ to provide phylogenetic analysis. In subsequent work, autumn descriptions were used for analyses related to microtopography, species prevalence, and species co-occurrence. In phylogenetic analyses of the flora, full lists of flora present at the studied sites were used.

The microtopography was measured using dynamic GSP (DGPS) Stonex-9 Plus equipment which allows sub-centimetre accuracy of measurements. The DGSP readings were taken with one meter interval. Maps of microtopography were calculated in SAGA GIS using Ordinary Kriging method. The resulting dataset for the ‘early’ site consisted of 86 microhighs and 225 microlows (microhighs had a relative height of 7–16 cm above the microlows), and the resulting dataset for the ‘late’ site consisted of 151 microhighs and 130 microlows (microhighs had a relative height of 17–40 cm above the microlows).

### Laboratory analysis and statistics

In soil samples, the electrical conductivity (EC) was measured in the lab with a 1:2.5 soil-to-water ratio using a Hanna Combo 98130 conductivity meter. In reference soil pits, located at typical microhighs and microlows, the moisture and soil bulk density were measured using the gravimetric method. All measures of EC as well as species composition of 2 × 2 plots are available as Supplementary Data.

Using published data, a list of 42 families from 22 orders of plants (Table S1) that grow on the Primorskaya lowland (in edaphic conditions typical for the key sites studied) was compiled (Tables S2, S3), as our key sites were located in the border between republics of Kalmykia and Dagestan (constituent entities of Russia; supplementary text 1).

Phylogenetic trees for Magnoliophyta species persistent in the ‘early’ or ‘late’ key sites and for families from the local flora were built based on a phylogenetic tree of Angiospermae from^41^. From either of the key sites, 27 of 35 species persisted in the phylogenetic tree of Angiospermae. Eight remaining species were added to the tree by adding their branches as basal polytomies within their genera or families with R scripts provided in^41^.

Taxonomic Distinctness Index based on presence-absence data was calculated as a mean pairwise taxonomic distance between species that were found in the community^42^.

Considering the two key sites as a proxy of time points along the chronosequence of the new terrestrial area, we investigated whether species that have been lost or acquired in the ‘late’ key site in compare with the ‘early’ site are randomly distributed, clustered or overdispersed across the evolutionary tree. The mean pairwise distance (MPD)—a measure that reflects the extent of phylogenetic clustering^25^—was calculated for species that were lost or acquired with time and compared with the null distributions for MPD as described in detail in our previous work^43^ and Supplementary text 2.

To determine whether species that had been lost in the ‘late' key site in comparison with the ‘early’ site preferably belonged to particular plant families, for each of 10 families of the ‘early’ site, the hypergeometric probability of losing the same or a greater number of species than were actually lost was counted under the null hypothesis that species were lost randomly.

To calculate the probability of four species acquired in the ‘late’ key site to belong to the same family Amaranthaceae by chance, the probability of each family to acquire a new species in the ‘late’ site was assumed to be proportional to its contribution to the species content of the ‘early’ site (fraction of the ‘early’ key site species belonging to this family). For Amaranthaceae, the hypergeometric probability of appearance of four new species or more was calculated under this null assumption.

Comparison of means (salinity [EC] and bare ground area) between microhighs and microlows at both key sites was performed with the nonparametric Mann–Whitney U-test.

All statistical analyses were performed with basic R language^44^. In a code for calculation of the taxonomic diversity index, “separate” function of “tidyr” package^45^ was used. Scripts that were written for this study are provided in Supplementary Folder 1 and on GitHub repository (https://github.com/GalkaKlink/CaspianSea) along with description of what they do.

## Results

### Distribution of Magnoliophyta families of the recent coastal lowland across evolutionary trees of local flora

The most abundant families in the dry steppes and deserts of the Caspian Sea region were Poaceae^6,46,47^ and Amaranthaceae. The last one includes the largest number of halophytes^48–50^. The ‘late’ site had 9 species of Amaranthaceae and 6 species of Poaceae, whereas the ‘early’ site had 6 species of Amaranthaceae and 10 species of Poaceae. Regarding the remaining eight families, the key sites had 1–3 (rarely 4) species belonging to them.

**Figure 1:**
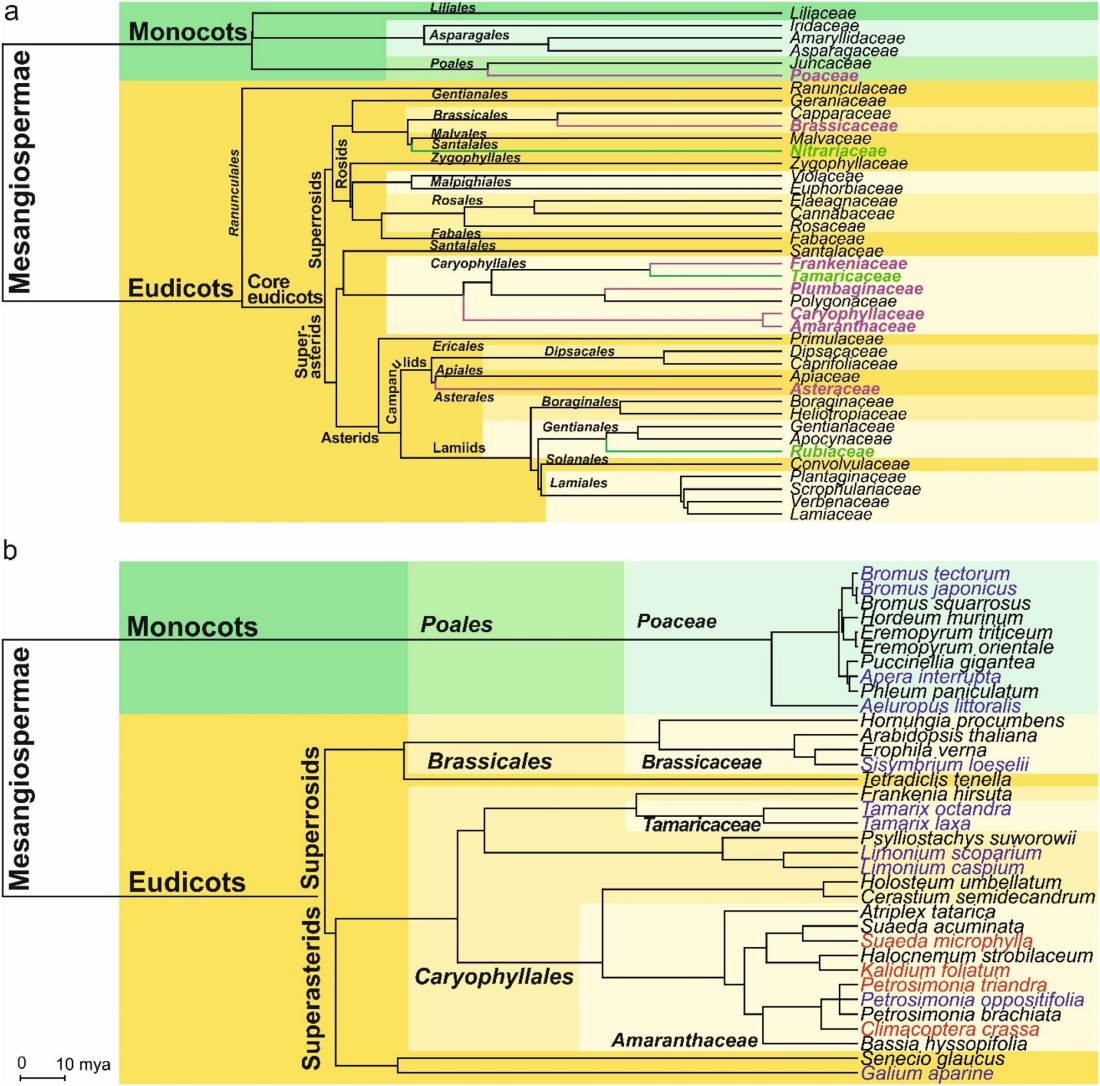
Phylogenetic tree of Magnoliophyta: (**a**) families growing in the western Caspian Sea cost in Dagestan and Kalmykia (purple—families that were found in both ‘early’ and ‘late’ sites, green—families that were found only in the ‘early’ site). (**b**) vegetation of the key sites (black—species that were found in both key sites, blue—species that were found only in the ‘early’ site, red—species that were found only in the ‘late’ site). Branch lengths are measured in millions of years.

Ten families from the ‘early’ key site belonged to six orders and were not significantly clustered on the evolutionary tree (p-value for MPD = 0.14). Half of the ‘early’ key site families (5 of 10) were from the order Caryophyllales, making this order significantly overrepresented (hypergeometric left p-value = 0.0004) among the pioneer vegetation during the draining of the Caspian Sea (Fig. 1a).

### Evolutionary analysis of species loss and gain

We have found 31 species in the Early site and 24 species in the Late site (Fig. 1b). The two key sites shared 20 species, which represented 65% and 83% of species found in the ’early’ and the ‘late’ sites, respectively. The flora of the two sites amounted to 35 species belonging to 10 families (Table S3). The number of common families was 8, which indicated a high degree of similarity of habitats in terms of family composition^29^.

We compared the diversity of two key sites by calculating the taxonomic distinctness index (TDI, see “Materials and methods”). Taxonomic diversity appeared to be similar for species that were found in the ‘early’ site, ‘late’ site, both sites and only in the ‘early’ site. However, it was twice smaller for species that were found only in the ‘late’ site, suggesting that this group consists of species that are closer related than species in other considered groups (Table 2).

**Table 2:** Species richness and taxonomic diversity of species that were found in the ‘early’ site and/or the ‘late’ site.

Group	Number of species	TDI
Early site	31	4.0
Late site	24	3.8
Both sites	20	4.0
Only early site	11	4.0
Only late site	4	2.0

**Figure 2:**
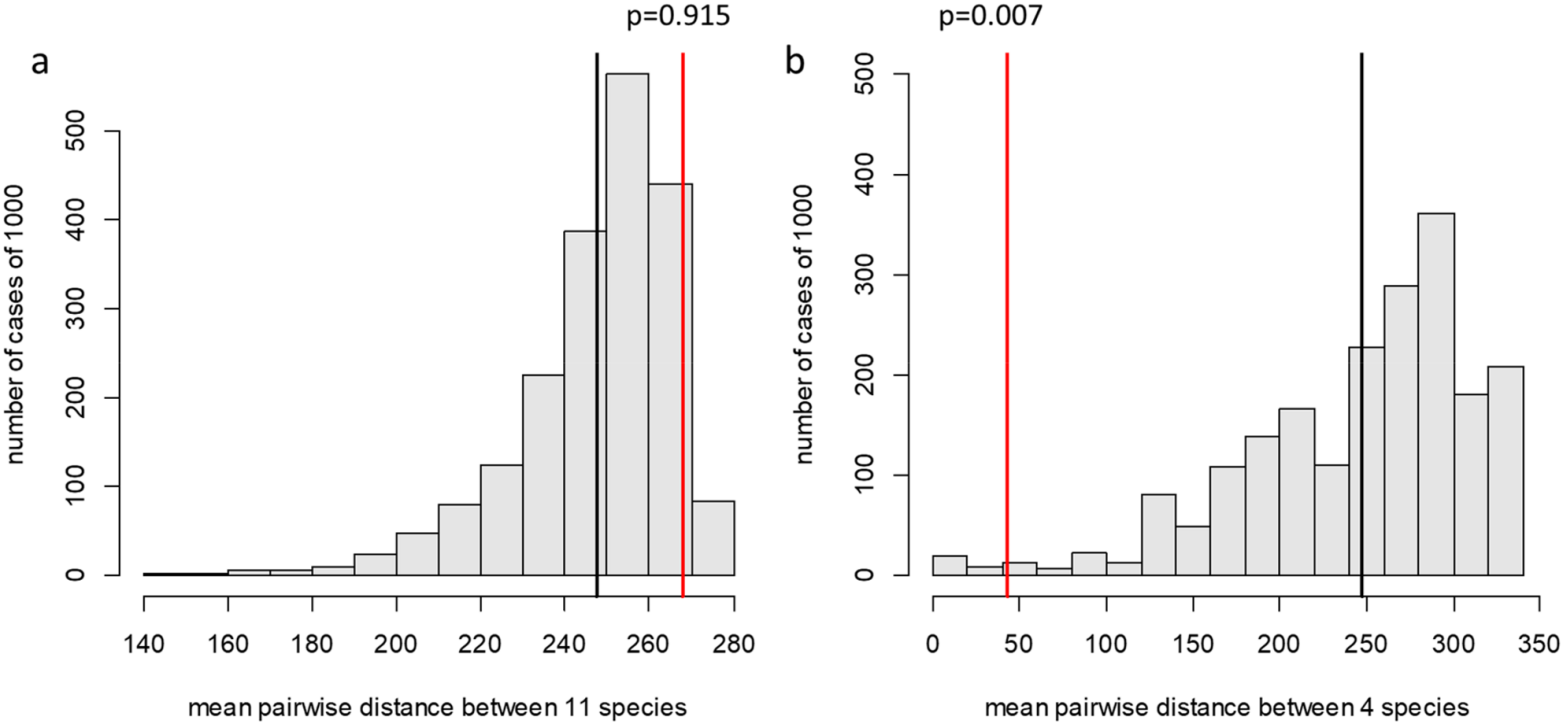
Null distributions for Mean Pairwise Distance (MPD) for 11 lost species (**a**) and 4 newly acquired species (**b**) in the ‘late’ site. The mean of each distribution is marked by a black line. MPD's of the lost (a) or newly acquired (b) species are marked by red lines. Above the red lines are the probabilities of occurrence of the observed or lower MPD for lost (**a**) or acquired (**b**) species in case of their random phylogenetic distribution.

Among 31 species of the ‘early’ key site, 11 species were absent in the ‘late’ site. However, four new species appeared in the ‘late’ site, with 24 species in total (Table S3). Species lost in the ‘late’ site were distributed randomly across the phylogenetic tree (p-value for phylogenetic clustering = 0.915), whereas acquired species were highly clustered (p-value for phylogenetic clustering = 0.007, Fig. 1b, Fig. 2). No families lost less or more species than was expected by chance (Table S4). As for acquired species, all four belonged to one family (Amaranthaceae), which was unlikely to occur by chance (p = 0.00047, see “Materials and methods”).

### Physical determinants of species loss and gain

The observed randomness of the phylogenetic distribution of species that were lost in the ‘late’ site could have occurred if the extinction was random. In such a case, the probability of a species to be lost would be proportional to its abundance in the ‘early’ key site. To test this, the abundance of each species in the ‘early’ site was estimated as a fraction of 2 × 2 plots, where this species was observed. Species that retained in the ‘late’ site were not more prevalent in the ‘early’ site than species that were lost in the ‘late’ site (one-sided Mann–Whitney test, p = 0.081), pointing that their extinction during succession was not random.

As salinity is considered the limiting factor for plant growth in the Caspian Sea coast, we tested whether differences in salinity between the two key sites may influence difference in their vegetation. Among several depth intervals (see “Materials and methods”), the percent of bare ground in 2 × 2 plots (both sites were considered together) showed the highest correlation with soil electroconductivity for a 30–50 cm layer (Spearman test, p = 0.0085, r = 0.3; Table S5). As root lengths of most species from the studied area fall within this interval (Table S3), it supports the importance of soil salinity for plant partitions in the studied area. For both microhighs and microlows, salinity was higher in the ‘late’ site than in the ‘early’ site for this soil layer (Table S6), suggesting that differences in salinity may be responsible, at least partially, for differences in vegetation coverage and composition between the two key sites.

To better understand the differences between 24 species that persisted in the 'late’ site and 11 species that persisted in the ‘early’ site but were absent in the ‘late’ site, two ecological characteristics of these species (an ability to live in a desert or being halophytic) were compared in binary form (yes/no) by Fisher’s exact test (Table S7). For spring descriptions, the ability to live in a desert (whether deserts are included in the species range, Table S3), a proxy of tolerance to water loss, was significantly overrepresented among species from the ‘late’ site (two-sided p = 0.001). Indeed, 83% of these species were desert plants in comparison with only 20% of species that were found only in the ‘early’ key site. Salt tolerance (whether the species is halophyte, Table S3) was not differentially represented (two-sided p = 1.00) among species found and lost in the ‘late’ key site, with 88% of persistent species and 90% of lost species. For autumn descriptions, desert species were no more overrepresented in the ‘late’ key site in comparison with plants from only the ‘early’ site, likely because the advantage of this feature becomes very high in both sites with the end of spring. Meanwhile, meadow plants became underrepresented in the ‘late’ site in autumn. Therefore, soil moisture and salinity might provide the differences in flora of the sites under consideration. In support of our assumption, three of four species that were met in the ‘late’ site but not in the ‘early’ site had a C4 type of photosynthesis (Table S3). C4-plants are known to be prevalent in arid environments and are believed to be more tolerant to increased soil salinity and water loss than plants with C3-type of photosynthesis^51–53^.

Along with abiotic factors, plant-plant interactions can also be responsible for differences in vegetation between the two sites. Large (> 1 m) tamarisk bushes seemed to influence the microtopographic structure and controlled the local microclimate around them. Tamarisk might serve as a nurse plant for some species in local patches. Therefore, some species could have been lost in the ‘late’ site due to tamarisk extinction. Among 10 species found in the ‘early’ key site in autumn, only *Limonium scoparium* occurred significantly more frequently in plots with large tamarisk bushes than in plots without large tamarisk (exact Fisher test, right-sided p-value = 0.003; Table S8), suggesting that tamarisk may serve as a nurse plant for this species and potentially for some ephemers, which is impossible to check with the available data.

### Influence of microtopography on vegetation of the two key sites

**Figure 3:**
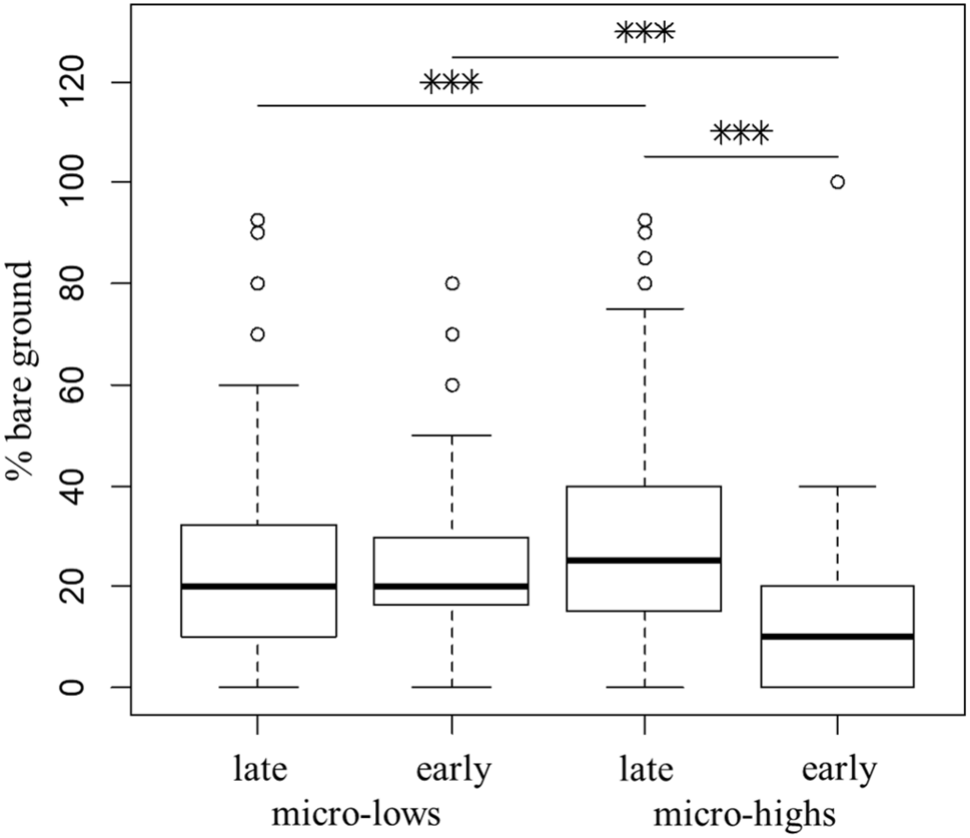
Percentage of bare ground in microhighs and microlows of the key sites. Bold line is the median. Box borders represent the interquartile range (IQR) between first (Q1) and third (Q3) quartiles. Whiskers are Q1 – 1.5*IQR and Q3 + 1.5*IQR. ***, Mann–Whitney’s two-sided p-value < 0.001.

In the ‘early’ site (Fig. 3), the mean percentage of bare ground was greater (Mann–Whitney’s two-sided test, p = 0.136 × 10^–6^) for microlows (26%) than for microhighs (16%). However, in the ‘late’ site, the situation was reversed, and microlows had smaller uncovered area than microhighs (25% vs 31%, p = 0.0073). When comparing the two key sites, microlows had a similar percentage of bare ground in both key sites (p = 0.07), but microhighs had twice as large uncovered ground area in the ‘late’ site than in the ‘early’ key site (p = 0.114 × 10^–6^, Fig. 3). Therefore, microhighs of the 'early’ site were the most vegetated, whereas microhighs of the ‘late’ site were the least covered.

To evaluate the microtopography preferences of plants, for each species, its prevalence in the 2 × 2 m plots from microhighs and microlows was compared using Fisher’s exact test at each key site. For tamarisk in the ‘early’ key site, small, medium, and large individuals were analysed separately.

In the ‘early’ key site, *Limonium scoparium* was overrepresented in microhighs, whereas *Petrosimonia brachiata* and *Suaeda acuminata* were overrepresented in microlows (Table 3). Interestingly, large tamarisk specimens were overrepresented in microhighs, whereas small specimens were slightly overrepresented in microlows.

**Table 3:** Differential prevalence of plant species between microhighs (“H”) and microlows (“L”).

Species	‘Late’ site				‘Early’ site			
	H	L	p-value*	Preferred**	H	L	p-value*	Preferred**
*Atriplex tatarica*	**0.007**	**0.076**	**0.002**	**L**	0.047	0.071	0.613	n.s.
*Bassia hyssopifolia*	0.007	0.025	0.241	n.s.	0.000	0.008	1.000	n.s.
*Climacoptera crassa*	**0.517**	**0.093**	**0.000**	**H**	–	–	–	–
*Frankenia hirsuta*	**0.026**	**0.490**	**0.000**	**L**	0.581	0.669	0.150	n.s.
*Halocnemum strobilaceum*	0.026	0.053	0.279	n.s.	0.000	0.004	1.000	n.s.
*Kalidium foliatum*	**0.351**	**0.612**	**0.000**	**L**	–	–	–	–
*Limonium caspium*	–	–	–	–	0.081	0.031	0.066	n.s.
*Limonium scoparium*	–	–	–	–	**0.174**	**0.047**	**0.001**	**H**
*Petrosimonia brachiata*	**0.901**	**0.622**	**0.000**	**H**	**0.419**	**0.674**	**0.000**	**L**
*Petrosimonia oppositifolia*	–	–	–	–	0.174	0.229	0.361	n.s.
*Suaeda acuminata*	0.026	0.091	0.014	L	0.012	0.101	0.005	L
*Suaeda microphylla*	0.947	0.912	0.256	n.s	–	–	–	–
Tamarisk big (> 1 m)	–	–	–	–	**0.744**	**0.238**	**0.000**	**H**
Tamarisk medium (0.5–1 m)	–	–	–	–	0.372	0.459	0.167	n.s.
Tamarisk small (< 0.5)	–	–	–	–	0.314	0.454	0.023	L
Tamarisk all	–	–	–	–	0.860	0.833	0.609	n.s.

For each species, the fraction of plots at which it was found (i.e. the number of plots where this species was found divided by the total number of plots) is shown.

*p-value of Fisher’s exact test. Species witht p-value < 0.05 after Bonferroni correction for multiple testing are in bold. Species for which the p-value was significant only before the correction are underlined.

**Preferred site according to Fisher’s exact test; n.s.—test is insignificant. H—microhighs. L—microlows. Dash—plant species did not occur at the key site.

In the ‘late’ site, *Climacoptera crassa* and *Petrosimonia brachiata* were overrepresented in microhighs, and *Atriplex tatarica*, *Frankenia hirsuta*, and *Suaeda acuminata* were overrepresented in microlows (Table 3). Among shrubs, *Kalidium foliatum* was overrepresented in microlows.

The prevalence of species at microhighs and microlows was also compared between the two key sites (Table 4). *Climacoptera crassa* preferred microhighs of the ‘late’ site. For *Petrosimonia brachiata*, microhighs of the ‘late’ site were the most preferable and microhighs of the ‘early’ site were the least preferable areas. *Frankenia hirsuta* was highly prevalent in all 2 × 2 m plots, except for those at microhighs of the ‘late’ site. It grew on more than 50% of plots at microlows of the ‘early’ and ‘late’ site, as well as microhighs of the ‘early’ site, but less than on 3% of plots at microhighs of the ‘late’ site. *Petrosimonia oppositifolia* and *Puccinellia gigantea* preferred the ‘early’ site over the ‘late’ site, irrespective of microtopography.

**Table 4:** Differential prevalence of plant species on microhighs and microlows between the key sites studied.

Species	Microhighs				Microlows			
	% of plots with species		p-value	Preferred key site	% of plots with species		p-value	Preferred key site
	Late site	Early site			Late site	Early site		
*Atriplex tatarica*	0.7	4.7	0.060	Early	12	8.0	0.343	Late
*Atriplex triticeum*	0	0	1.000	None	0.8	0	0.366	Late
*Bassia hyssopifolia*	0.7	0	1.000	Late	3.8	0.9	0.105	Late
*Climacoptera crassa*	52	0	**0.000**	Late	12	0	**0.000**	Late
*Frankenia hirsuta*	2.6	58	**0.000**	Early	73	72	0.902	Late
*Halocnemum strobilaceum*	2.6	0	0.300	Late	7.7	0	**0.000**	Late
*Halocnemum strobilacium*	0	0	1.000	None	0	0.4	1.000	Early
*Kalidium foliatum*	35	0	**0.000**	Late	72	0	**0.000**	Late
*Limonium caspium*	0	8.1	**0.001**	Early	0	3.6	**0.029**	Early
*Limonium scoparium*	0	17	**0.000**	Early	0	5.3	**0.005**	Early
*Petrosimonia brachiata*	89	42	**0.000**	Late	68	72	0.397	Early
*Petrosimonia oppositifolia*	0	17	**0.000**	Early	0	25	**0.000**	Early
*Puccinellia gigantea*	0	84	**0.000**	Early	0	74	**0.000**	Early
*Suaeda acuminata*	2.6	1.2	0.656	Late	14	12	0.616	Late
*Suaeda mycrophylla*	88	0	**0.000**	Late	89	0	**0.000**	Late
*Tamarix* sp.	0	86	**0.000**	Early	0	87	**0.000**	Early

Species with Exact Fisher’s test p-value < 0.05 after Bonferroni correction for multiple testing are in bold.

Therefore, plant preferences depended complexly on microtopography, as well as area age. In the ‘early’ site, salinity depended on microtopography at a depth of 0–30 cm and was higher for microlows, whereas in the ‘late’ key site, microhighs and microlows differed in salinity only at a depth of 50–100 cm (Table S6). Given that 78% of species in the ‘early’ site and 88% of species at the ‘late’ site had a maximal root length of no more than 50 cm (Table S3), it suggests that the importance of soil salinity for differences in vegetation between plots is higher in the ‘early’ site than in the ‘late’ site.

## Discussion

As the Caspian Sea gradually retreats, lands of different ages forming a chronosequence are available for research. In this paper, the system of two key sites that appeared from the Caspian Sea 1412 and 365 years ago were considered as a proxy of two time points to estimate how local plant communities appear and transform while the sea is drying. Such a technique of space-for-time substitution is frequently used in comparative studies of territories with different level of disturbance or different times since disturbance^54^. Although use of chronosequences is indispensable in case of long-term scales (as it is for our case), it may lead to wrong conclusions when some factors other than time have considerable influence on the differences between studied sites^55^. However, as our key sites are located less than in 2 km from each other, they experience similar influence of climatic factors and are available for colonization by the same plants. As the two key sites appeared from the sea with interval about 1000 years, their initial climatic conditions potentially might be different. However, the studied area has remained semi-arid for the 3000 years^20^. In this work, we were interested in transformation of young dried areas. Therefore, only two sites were considered. However, it will be interesting to consider the whole chronosequence covering 3000 years of the history of the Caspian Sea retreat in semi-arid conditions.

A decrease in species richness (number of species) as the sea retreats and the newly formed land becomes drier was previously recorded in studies of the Aral Sea region^56^. The previous work on the ‘early’ and ‘late’ key sites showed a decrease in species richness in the latter in comparison with the former. Meanwhile, the communities of the key sites were similar in terms of the number of biomorphotypes and annual herbs^29^. Here, using taxonomic diversity index, we found that despite differences in species richness, phylogenetic diversity was similar between the two key sites.

Our evolutionary analysis of species that were unique for either of the two key sites showed that species loss in the ‘late’ site in comparison with the ‘early’ site occurred irrespective of evolutionary relationships, whereas acquired species were phylogenetically clustered. Therefore, we suggest that different plant traits are important to survive and appear de novo in dynamic communities forming in the changing environment of the Caspian Sea coast. Traits that were important for staying in a community did not show a pronounced phylogenetic signal, and different plants may survive due to different combinations of traits. In contrast, the set of features important for coming into a community seems to be narrow and to have a strong phylogenetic signal on the evolutionary tree of the local flora. One may speculate that it is good to be a generalist to survive in changing conditions of the Caspian Sea coast, whereas it is necessary to be a specialist to break into the existing but stressed community. All species growing in the ‘late’ site but absent in the ‘early’ site belonged to the Amaranthaceae family, which is known to increase in proportion with the climate desertification in steppes^57–60^.

Our results suggest that microtopography has a noticeable impact on vegetation in both key sites. In the ‘early’ key site, the salinity of root-inhabited soil layers was lower for microhighs than for microlows, where the percentage of bare ground was greater contrary to our hypothesis. Therefore, for the ‘early’ key site, soil salinity was likely a determinant of better growing conditions at microhighs than at microlows. Additionally, big tamarisk bushes growing at microhighs of the ‘early’ site (and potentially forming them^21^) may serve as a nurse plant for some other plant species. The increase in the role of positive plant-plant interactions was previously shown for stressed environments^61,62^.

In the ‘late’ key site, microlows were better populated than microhighs. In the same time, the difference in soil salinity between microlows and microhighs was insignificant for root-inhabited layer, suggesting that factors other than salinity provide the difference in occupancy of microtopographic elements in this key site. Interestingly, *Petrosimonia brachiata* was significantly more abundant at microlows of the ‘early’ site and microhighs of the ‘late’ site, making it a potential indicator for highly stressed parts of the environment. *Petrosimonia* spp. were previously found to live in soils with salinity of 2 to 28 dS m^–1^^50,63^.

As the ability to live in deserts appeared to be important for growth in the ‘late’ site, we suggest that soil moisture determines the composition of the plant community in the ‘late’ site. Disappearing of tamarisk in the ‘late’ key site may also suggest this, as tamarisk was shown to drop out of the community with the decrease in groundwater level in tugay forests of Middle Asia^3^. Additionally, the Chenopodioideae/Asteraceae ratio was 1.5 times higher for the ‘late’ site than for the ‘early’ site (9/1 vs 6/1, respectively), which indicates a more arid environment^64,65^. Therefore, species growing at the ‘late’ key site tended to be more tolerant to water loss than species growing at the ‘early’ site.

## Conclusion

In the studied system, plant preferences were complexly correlated with microtopography and area age. Our hypothesis suggesting that for the studied area, the environment for plants is better in elevated areas than in lowlands was confirmed on macrolevel. However, on the level of microtopography it was confirmed only for elder site, whereas for the ‘early’ site our results suggest the opposite.

The phylogenetic distribution of species that were lost in the ‘late’ site in comparison with ‘early’ site was random, whereas species that appeared in the ‘late’ site but were absent in the ‘early’ site were highly clustered on a phylogenetic tree, suggesting that vulnerability to a changing environment depends on traits that do not show a phylogenetic signal, but the ability to appear in community de novo requires properties possessed by the Amaranthaceae family. Mosaic patches of vegetation had a pronounced correlation with microtopography. Edaphic conditions of microhighs and microlows changed with time, leading to changes in the spatial distribution of plants.

Our work demonstrates the importance of considering both evolutionary and ecological plant traits in studying plant community succession at the Caspian Sea coast.

## Supplementary Information

Supplementary Information.

## Data Availability

The data that supports the findings of this study is available in the Supplementary Data of this article. The data on one of the two key sites was published (Semenkov et al.^30^; 10.1016/j.dib.2020.105972). Voucher specimens are deposited in a publicly available herbarium of the Tsytsyn Main Botanical Garden of the Russian Academy of Sciences (the deposition number of all plant species collected: *Tetradiclis tenella*, MHA 0250435; *Hymenolobus procumbens*, MHA0269285; *Halocnemum strobilaceum*, MHA0269286; *Suaeda microphylla*, MHA0269287; *Bassia hyssopifolia*, MHA0269288; *Kalidum foliatum*, MHA0269289; *Frankenia hirsuta*, MHA0269290; *Petrosimonia brachiata*, MHA0269291; *Petrosimonia oppositifolia*, MHA0269292; *Tamarix octandra*, MHA0269293; *Tamarix laxa*, MHA0269294; *Puccinellia gigantea*, MHA0269295; *Limonium scoparium*, MHA0269296; *Senecio noeanus*, MHA0269297).

## Code availability

All statistical procedures and data formatting was performed with basic functions. Perl and R scripts are available on GitHub https://github.com/GalkaKlink/CaspianSea.
