# Supplementary material for: Temporal change in plant communities and its relationship to soil salinity and microtopography on the Caspian Sea coast
Source: Sci Rep. 2022 Oct 27;12:18082. doi: 10.1038/s41598-022-19863-5 (PMC9614000; doi:10.1038/s41598-022-19863-5)
Supplement: Supplementary file 1 — Supplementary Information. [file 41598_2022_19863_MOESM1_ESM.docx]

# SUPPLEMENTARY MATERIALS

# Temporal change in plant communities and its relationship to soil salinity and microtopography on the Caspian Sea coast

Galya V. Klink^a^, Ivan N. Semenkov^b,*,^, Yulia D. Nukhimovskaya^c^, Zarema Ul. Gasanova^d^, Nina Yu. Stepanova^e^, Maria V. Konyushkova^b^

^a^ Institute for Information Transmission Problems (Kharkevich Institute) of the Russian Academy of Sciences, Moscow 127051, Russia

^b^ Lomonosov Moscow State University, Moscow 119991, Russia

^c^ Severtsov Institute of Ecology and Evolution of the Russian Academy of Sciences, Moscow 119071, Leninskii pr. 33, Russia

^d^ Precaspian Institute of Biological Resources of the Daghestan Federal Research Centre of the Russian Academy of Sciences, Makhachkala 367000, Russia

^e^ Tsytsyn Main Botanical Garden of the Russian Academy of Sciences, Moscow 127276, Russia

*Corresponding author: semenkov@geogr.msu.ru


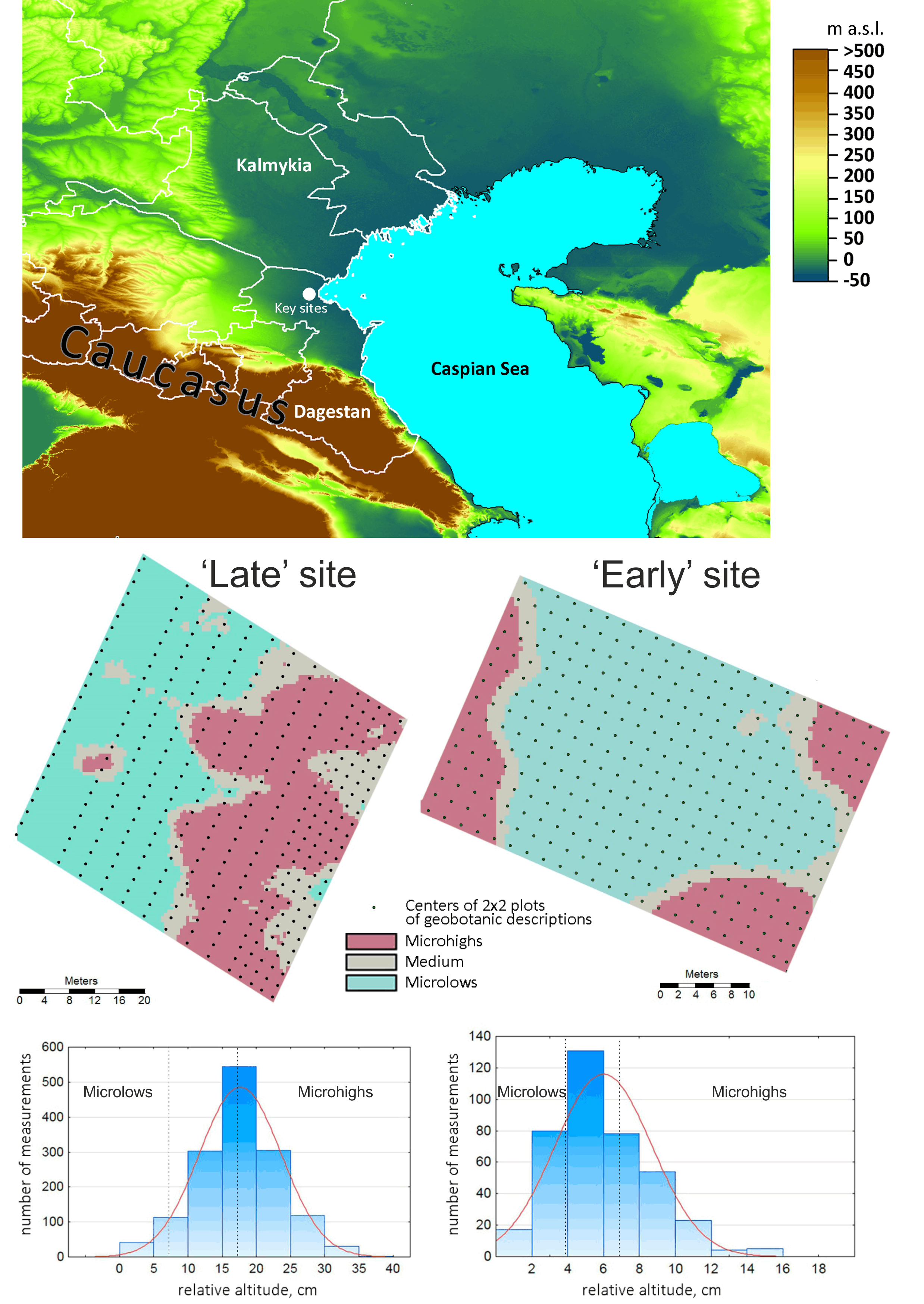


Figure S 1. Key area location (up), microtopography of the key sites (middle) and histograms of relative elevations (down). All maps are prepared by M.V. Konyushkova in the SAGA GIS software, version 8.2.1; https://sourceforge.net/projects/saga-gis/files/

### Supplementary tables

#### Table S 1. List of Magnoliophyta families typical for Primorskaya lowlands in Dagestan and Kalmykia

| Family | Order | Dagestan | | Kalmykia | | Key sites | |
| --- | --- | --- | --- | --- | --- | --- | --- |
|  |  | ^1^ | ^2^ | ^3^ | ^4^ | ‘late’ | ‘early’ |
| Amaryllidaceae | Asparagales | No | Yes | Yes | Yes | No | No |
| Apiaceae | Apiales | Yes | Yes | Yes | Yes | No | No |
| Apocynaceae | Gentianales | Yes | Yes | Yes | Yes | No | No |
| Asparagaceae | Asparagales | Yes | Yes | Yes | Yes | No | No |
| Asteraceae | Asterales | Yes | Yes | Yes | Yes | Yes | Yes |
| Boraginaceae | Boraginales | Yes | Yes | Yes | Yes | No | No |
| Brassicaceae | Brassicales | Yes | Yes | Yes | Yes | Yes | Yes |
| Cannabaceae | Rosales | No | Yes | Yes | Yes | No | No |
| Capparaceae | Brassicales | Yes | Yes | No | No | No | No |
| Caryophyllaceae | Caryophyllales | Yes | Yes | Yes | Yes | Yes | Yes |
| Amaranthaceae | Caryophyllales | Yes | Yes | Yes | Yes | Yes | Yes |
| Convolvulaceae | Solanales | Yes | Yes | Yes | Yes | No | No |
| Dipsacaceae | Dipsacales | Yes | Yes | No | Yes | No | No |
| Elaeagnaceae | Rosales | Yes | Yes | No | No | No | No |
| Equisetaceae | Equisetales | No | Yes | No | No | No | No |
| Euphorbiaceae | Malpighiales | Yes | Yes | Yes | Yes | No | No |
| Fabaceae | Fabales | Yes | Yes | Yes | Yes | No | No |
| Frankeniaceae | Caryophyllales | Yes | Yes | Yes | Yes | Yes | Yes |
| Gentianaceae | Gentianales | Yes | Yes | Yes | No | No | No |
| Geraniaceae | Geraniales | Yes | Yes | Yes | Yes | No | No |
| Heliotropiaceae | Boraginales | Yes | Yes | No | No | No | No |
| Iridaceae | Asparagales | Yes | Yes | Yes | Yes | No | No |
| Juncaceae | Poales | Yes | Yes | Yes | Yes | No | No |
| Lamiaceae | Lamiales | Yes | Yes | Yes | Yes | No | No |
| Liliaceae | Liliales | No | Yes | Yes | Yes | No | No |
| Malvaceae | Malvales | Yes | Yes | Yes | Yes | No | No |
| Nitrariaceae | Sapindales | Yes | Yes | Yes | Yes | No | Yes |
| Plantaginaceae | Lamiales | Yes | Yes | Yes | Yes | No | No |
| Plumbaginaceae | Caryophyllales | Yes | No | No | Yes | Yes | Yes |
| Poaceae | Poales | Yes | Yes | Yes | Yes | Yes | Yes |
| Polygonaceae | Caryophyllales | Yes | Yes | Yes | Yes | No | No |
| Primulaceae | Ericales | Yes | Yes | Yes | Yes | No | No |
| Ranunculaceae | Ranunculales | Yes | Yes | No | Yes | No | No |
| Rosaceae | Rosales | Yes | Yes | Yes | Yes | No | No |
| Rubiaceae | Gentianales | No | Yes | Yes | Yes | No | Yes |
| Santalaceae | Santalales | No | Yes | Yes | Yes | No | No |
| Scrophulariaceae | Lamiales | Yes | Yes | Yes | Yes | No | No |
| Tamaricaceae | Caryophyllales | Yes | Yes | Yes | Yes | Yes | Yes |
| Caprifoliaceae | Dipsacales | Yes | No | Yes | Yes | No | No |
| Verbenaceae | Lamiales | No | Yes | Yes | No | No | No |
| Violaceae | Malpighiales | No | Yes | No | Yes | No | No |
| Zygophyllaceae | Zygophyllales | Yes | Yes | Yes | Yes | No | No |

#### Table S 2. Brief description of the references used to compile the list of the local flora of loamy habitats at the Primorskaya lowland in Dagestan and Kalmykia with a groundwater level of 2–2.5 m

| Region | | N of families of Magnoliophyta | Brief description | Reference |
| --- | --- | --- | --- | --- |
| Dagestan | All | 154 | All plant species observed at Dagestan | ^5–8^ |
|  | Primorskaya lowland* | 101 | Data on 2,000 herbarium samples of halophytes and other plant groups | ^9^ |
|  | Primorskaya lowland at the Dagestan’s Caspian Sea coast* from Makhachkala in the north to the Samur River delta in the south and from the Caspian Sea coast in the east to the foothills of the Caucasus | 100 | Review on location and ecology of species | ^2,5–8^ |
|  | Site "Kizlyar Bay" of the Dagestan Reserve | 50 | All plant species observed | ^10^ |
| Kalmykia | All | 84 | All plant species observed at Kalmykia. | ^4^ |
|  | Eastern part | 57 | Flora of the four key sites located at the Novo-Caspian, late and early Khvalynian plains, as well as on the border of the early and late Khvalynian plains. | ^3^ |

* 160 km length from north to south, 10-40 km width from west to east

#### Table S 3. Selected proxies of the flora of the studied sites

| Species | | Family ^11^ | Biomorphotype (life form) | Pathway of photosynthesis ^12–26^ | Key site | | Maximum depth of root penetration, cm | Ecomorphotype | | Ecological-cenotic group ^3,27^ | Specialist | Voucher specimen ID* |
| --- | --- | --- | --- | --- | --- | --- | --- | --- | --- | --- | --- | --- |
| ^28^ | ^29,30^ |  |  |  | ‘early’ | ‘late’ |  | salinization | soil moisture |  |  |  |
| Aeluropus littoralis | Aeluropus littoralis | Poaceae | Long-lived perennial herbs | C4 | yes | no | 50T | H | XM | H-M-S | YN | n.a. |
| Anisantha tectorum | Bromus tectorum | Poaceae | Ephemeral | C3 | yes | no | 30O | GH | M | R | YN | n.a. |
| Apera interrupra | Apera interrupta | Poaceae | Ephemeral | ? | yes | no | 30O | HG | M | H-M-S | YN | n.a. |
| Arabidopsis thaliana | Arabidopsis thaliana | Brassicaceae | Ephemeral | C3 | yes | yes | 30O | HG | XM | S | YN | n.a. |
| Atriplex tatarica | Atriplex tatarica | Amaranthaceae | Annual herbs | C4 | yes | yes | 50T | GH | MX | R | YN | n.a. |
| Bassia hyssopifolia | Bassia hyssopifolia | Amaranthaceae | Annual herbs | C4 | yes | yes | 50T | H | MX | D-S | YN | MHA0269288 |
| Bromus japonicus | Bromus japonicus | Poaceae | Ephemeral | C3 | yes | no | 30O | HG | M | R | YN | n.a. |
| Bromus squarrosus | Bromus squarrosus | Poaceae | Ephemeral | ? | yes | yes | 30O | HG | XM | S | YN | n.a. |
| Cerastium semidecandrum | Cerastium semidecandrum | Caryophyllaceae | Ephemeral | ? | yes | yes | 30O | G | M | P-S | YN | n.a. |
| Climacoptera crassa | Climacoptera crassa | Amaranthaceae | Annual herbs | C4 | no | yes | 50T | HH | X | D | YN | n.a. |
| Eremopyron orientale | Eremopyrum orientale | Poaceae | Ephemeral | C3 | yes | yes | 30O | GH | X | D-S | YN | n.a. |
| Eremopyron triticeum | Eremopyrum triticeum | Poaceae | Ephemeral | C3 | yes | yes | 30O | GH | X | D-S | YN | n.a. |
| Erophila verna | Erophila verna | Brassicaceae | Ephemeral | ? | yes | yes | 10O | G | M | P-S | YN | n.a. |
| Frankenia hirsuta | Frankenia hirsuta | Frankeniaceae | Subshrubs | C3 | yes | yes | 80T | HH | MH | D-H | YN | MHA0269290 |
| Galium aparine | Galium aparine | Rubiaceae | Ephemeral | C3 | yes | no | 30O | G | M | R | YN | n.a. |
| Halocnemum strobilaceum | Halocnemum strobilaceum | Amaranthaceae | Long-lived perennial herbs | C3 | yes | yes | 200F | HH | X | D-H | YN | MHA0269286 |
| Holosteum glutinosum | Holosteum umbellatum | Caryophyllaceae | Ephemeral | ? | yes | yes | 30O | GH | M | D | YN | n.a. |
| Hordeum leporinum | Hordeum murinum | Poaceae | Ephemeral | C3 | yes | yes | 30O | HG | MX | R | YN | n.a. |
| Hymenolobus procumbens | Hornungia procumbens | Brassicaceae | Ephemeral | ? | yes | yes | 30O | HG | M | D-H | YN | MHA0269285 |
| Kalidum foliatum | Kalidum foliatum | Amaranthaceae | Subshrubs | C3 | no | yes | 300F | HH | X | D-H | YN | MHA0269289 |
| Limonium caspium | Limonium caspium | Plumbaginaceae | Long-lived perennial herbs | C3 | yes | no | 200F | HH | MH | H-M-S | YN | n.a. |
| Limonium scoparium | Limonium scoparium | Plumbaginaceae | Long-lived perennial herbs | C3 | yes | no | 300F | H | X | D-P | YN | MHA0269296 |
| Petrosimonia brachiata | Petrosimonia brachiata | Amaranthaceae | Annual herbs | C4 | yes | yes | 50T | H | X | D-S | YN | MHA0269291 |
| Petrosimonia oppositifolia | Petrosimonia oppositifolia | Amaranthaceae | Annual herbs | C4 | yes | no | 50T | H | X | D-S | YN | MHA0269292 |
| Petrosimonia triandra | Petrosimonia triandra | Amaranthaceae | Annual herbs | C4 | no | yes | 50T | H | X | H-M-S | YN | n.a. |
| Phleum paniculatum | Phleum paniculatum | Poaceae | Ephemeral | C3 | yes | yes | 30O | G | XM | S | YN | n.a. |
| Psylliostachys spicata | Psylliostachys suworowii | Plumbaginaceae | Annual herbs | ? | yes | yes | 30O | H | MX | D-S | YN | n.a. |
| Puccinellia gigantea | Puccinellia gigantea | Poaceae | Long-lived perennial herbs | ? | yes | yes | 80T | H | M | H-M-S | YN | MHA0269295 |
| Senecio noeanus | Senecio glaucus | Asteraceae | Ephemeral | ? | yes | yes | 30O | HG | XM | D | YN | MHA0269297 |
| Sisymbrium loeselii | Sisymbrium loeselii | Brassicaceae | Ephemeral | C3 | yes | no | 30O | HG | MX | R | YN | n.a. |
| Suaeda acuminata | Suaeda acuminata | Amaranthaceae | Annual herbs | C4 | yes | yes | 50T | HH | XM | D-H | YN | n.a. |
| Suaeda microphylla | Suaeda microphylla | Amaranthaceae | Subshrubs | C4 | no | yes | 350F | HH | X | D-H | YN | MHA0269287 |
| Tamarix laxa | Tamarix laxa | Tamaricaceae | Shrubs | ? | yes | no | 250F | H | XM | D-H | YN, NS | MHA0269294 |
| Tamarix octandra | Tamarix octandra | Tamaricaceae | Shrubs | ? | yes | no | 250F | H | XM | D-H | YN, NS | MHA0269293 |
| Tetradiclis tenella | Tetradiclis tenella | Nitrariaceae | Ephemeral | ? | yes | yes | 10O | GH | XM | D-H | NS | MHA 0250435 |

To indicate the depth of root penetration, we used ^31–35^. The attitude to the source of moisture was estimated based on the works and concepts on the life forms of plants in other regions and conditions: F – phreatophyte, T – trichohydrophyte, O – ombrophyte. Ecomorphotype as based on salinization: G – Glycophyte, GH – Glycohalophyte, HG – Haloglycophyte, HH – Hyperhalophyte. Ecomorphotype as based on soil moisture: M – Mesophyte, MH – Mesohygrophyte, MX – Mesoxerophyte, XM – Xeromesophyte, X – Xerophyte ^3,27,36–38^. Ecological-cenotic group: D – Desert, H – Halophilic, M – Meadowy, P – Psammophilic, R – Ruderal, S – Steppe. Specialist is who undertook the formal identification of the plant material (YN – Yulia Nukhimovskaya, NS – Nina Stepanova). * a deposition number at the Tsytsyn Main Botanical Garden of the Russian Academy of Sciences. n.a. – not available (plant samples were not collected).

#### Table S 4. Significance values for the over- and underrepresentation of plant families among species lost in the ‘late’ key site

| Family | ’Early’ site species | | p-value | |
| --- | --- | --- | --- | --- |
|  | all | lost in the 'late’' site | family over-representation | family under-representation |
| Amaranthaceae | 6 | 1 | 0.947 | 0.284 |
| Asteraceae | 1 | 0 | 1.000 | 0.645 |
| Brassicaceae | 4 | 1 | 0.846 | 0.553 |
| Caryophyllaceae | 2 | 0 | 1.000 | 0.409 |
| Frankeniaceae | 1 | 0 | 1.000 | 0.645 |
| Nitrariaceae | 1 | 0 | 1.000 | 0.645 |
| Plumbaginaceae | 3 | 2 | 0.281 | 0.963 |
| Poaceae | 10 | 4 | 0.510 | 0.779 |
| Rubiaceae | 1 | 1 | 0.355 | 1.000 |
| Tamaricaceae | 2 | 2 | 0.118 | 1.000 |

#### Table S 5. Spearman’s rank correlation of the percentage of bare ground in 2×2 m plots with EC at different depths. The only correlation with a p-value < 0.1 after Bonferroni correction is shown in bold

| Soil layer, cm | r | p-value | N |
| --- | --- | --- | --- |
| 0–5 | 0,239 | 0,039 | 75 |
| 5–10 | 0,172 | 0,139 | 75 |
| 10–20 | 0,166 | 0,154 | 75 |
| 20–30 | 0,160 | 0,170 | 75 |
| **30–50** | **0,302** | **0,009** | **75** |
| 50–70 | 0,196 | 0,094 | 74 |
| 70–100 | -0,007 | 0,952 | 75 |

#### Table S 6. Mann-Whitney test for comparison of soil EC between microhighs (H) and microlows (L) at the ‘late’ and ‘early’ key sites

| Depth, cm | Mean salinity, dS/m | | p-value | Bonferroni | Number of plots at the key site | | Key site with higher salinity | Mean salinity, dS/m | | p-value | p-value after Bonferroni correction | Number of plots | | Plot with higher salinity |
| --- | --- | --- | --- | --- | --- | --- | --- | --- | --- | --- | --- | --- | --- | --- |
|  | ‘late’ | ‘early’ |  |  | ‘late’ | ‘early’ |  | H | L |  |  | H | L |  |
| Microhighs | | | | | | | | ‘Late’ key site | | | | | | |
| 0–5 | 11.6 | 7.2 | 0.055 | 0.382 | 13 | 18 | C1o | 11.6 | 16.9 | 0.566 | 3.964 | 13 | 5 | L |
| 5–10 | 12.1 | 7.5 | 0.026 | 0.184 | 13 | 18 | C1o | 12.1 | 12.9 | 0.521 | 3.647 | 13 | 5 | L |
| **10–20** | **11.9** | **7.3** | **<0.001** | **0.001** | 13 | 18 | C1o | 11.9 | 12.5 | 0.323 | 2.261 | 13 | 5 | L |
| **20–30** | **12.1** | **8.5** | **0.002** | **0.015** | 13 | 18 | C1o | 12.1 | 12.7 | 0.621 | 4.350 | 13 | 5 | L |
| **30–50** | **13.8** | **10.0** | **<0.001** | **0.001** | 13 | 18 | C1o | 13.8 | 13.9 | 0.961 | 6.724 | 13 | 5 | L |
| **50–70** | **12.7** | **9.8** | **<0.001** | **0.001** | 13 | 18 | C1o | 12.7 | 10.4 | 0.008 | 0.054 | 13 | 5 | H |
| 70–100 | 10.0 | 10.2 | 0.904 | 6.330 | 13 | 18 | C2y | **10.0** | **12.2** | **0.004** | **0.030** | 13 | 5 | L |
| Microlows | | | | | | | | ‘Early’ key site | | | | | | |
| 0–5 | 16.9 | 13.8 | 0.509 | 3.566 | 5 | 28 | C1o | 7.2 | 13.8 | 0.008 | 0.055 | 18 | 28 | L |
| 5–10 | 12.9 | 11.9 | 0.651 | 4.557 | 5 | 28 | C1o | **7.5** | **11.9** | **0.001** | **0.006** | 18 | 28 | L |
| 10–20 | 12.5 | 9.6 | 0.024 | 0.166 | 5 | 28 | C1o | **7.3** | **9.6** | **0.005** | **0.037** | 18 | 28 | L |
| 20–30 | 12.7 | 11.1 | 0.209 | 1.464 | 5 | 28 | C1o | **8.5** | **11.1** | **0.001** | **0.007** | 18 | 28 | L |
| **30–50** | **13.9** | **10.1** | **0.001** | **0.008** | 5 | 28 | C1o | 10.0 | 10.1 | 0.485 | 3.395 | 18 | 28 | L |
| 50–70 | 10.4 | 10.2 | 0.615 | 4.307 | 5 | 28 | C1o | 9.8 | 10.2 | 0.528 | 3.698 | 18 | 28 | L |
| 70–100 | 12.2 | 11.2 | 0.119 | 0.834 | 5 | 28 | C1o | 10.2 | 11.2 | 0.019 | 0.130 | 18 | 28 | L |

Tests with p-value < 0.05 after Bonferroni correction are shown in bold

#### Table S 7. Testing the hypothesis that certain traits were overrepresented or underrepresented among plants found at the ‘late’ key site or only at the ‘early’ key site; Fisher’s exact test

| Traits | Spring | | | Autumn | | |  |
| --- | --- | --- | --- | --- | --- | --- | --- |
|  | Fisher test p-value | fraction of species found at | | Fisher test p-value | fraction of species found at | | |
|  |  | ‘late’ site | ‘late’ site only |  | ‘late’ site | ‘late’ site only | |
| Freatophyte | 0.328 | 0.125 | 0.300 | 1.000 | 0.333 | 0.429 | |
| Galophyte | 1.000 | 0.875 | 0.900 | 1.000 | 1.000 | 1.000 | |
| Desert | **0.001** | **0.833** | **0.200** | 0.106 | 0.889 | 0.429 | |
| Steppe | 1.000 | 0.500 | 0.500 | 0.041 | 0.222 | 0.857 | |
| Meadow | 0.048 | 0.083 | 0.400 | **0.019** | **<0.001** | **0.571** | |
| C4 | 0.692 | 0.292 | 0.200 | 0.615 | 0.667 | 0.429 | |
| N of species | - | 24 | 10 | - | 9 | 7 | |

#### Table S 8. Fisher’s exact test to check the tendency of species to be associated with large tamarisk bushes at the ‘early’ key site

| Species | Number of plots | | | | Fisher’s exact test p-value | | Occurrence at the ‘late’ key site |
| --- | --- | --- | --- | --- | --- | --- | --- |
|  | Species (o) | | Species (a) | |  |  |  |
|  | tamarisk  (o) | tamarisk  (a) | tamarisk  (o) | tamarisk  (a) | association | anti-association |  |
| Atriplex tatarica | 5 | 22 | 126 | 192 | 0.994 | 0.021 | Y |
| Bassia hyssopifolia | 0 | 2 | 131 | 212 | 1.000 | 0.384 | Y |
| Eremopyrum triticeum | 1 | 0 | 130 | 214 | 0.380 | 1.000 | Y |
| Frankenia hirsuta | 86 | 144 | 45 | 70 | 0.668 | 0.421 | Y |
| Halocnemum strobilaceum | 0 | 1 | 131 | 213 | 1.000 | 0.620 | Y |
| Limonium caspium | 11 | 8 | 120 | 206 | 0.057 | 0.980 | N |
| **Limonium scoparium** | **19** | **11** | **112** | **203** | **0.003** | **0.999** | **N** |
| Petrosimonia brachiata | 68 | 148 | 63 | 66 | 1.000 | 0.001 | Y |
| Petrosimonia oppositifolia | 23 | 59 | 108 | 155 | 0.989 | 0.022 | N |
| Suaeda acuminata | 3 | 25 | 128 | 189 | 1.000 | 0.001 | Y |

Test with significant association (p-value < 0.05) after Bonferroni correction is shown in bold. Y—yes, N—no, (o) – occurrence, (a) – absence

### Supplementary text 1. Compilation of a local flora list

Using published data, a list of 42 families from 22 orders (Table S 1) of plants that grow on the Primorskaya lowland (in edaphic conditions typical for the key sites studied) in Dagestan ^1,2,5–9^ and Kalmykia ^3,4^ was compiled, as our key sites were located in the border between these two constituent entities of Russia. For Kalmykia and Dagestan, 38 families were common, and 4 families were unique to Dagestan. To compile a list of the Magnoliophyta flora of the loamy habitats at Primorskaya lowland with a groundwater level of 2–3 m from the primary sources (Table S 2), families whose species were not observed in similar territories or belonged to one of the group were removed: obligate psammophytes; obligate hydrophytes or hygrophytes, including growing exclusively in estuaries or paddy soils; weed and cultural plant species associated exclusively with roads, gardens, arable lands, and human settlements; inhabiting only the steppe part of Kalmykia or Dagestan; shrubs and trees that live only in forests; broomrapes affecting wormwood, weeds, and invasive species typical for the dry territories of the Caspian lowland.

As a rule, rejected families were represented in the original flora lists by one or two species. In controversial cases (for Cannabaceae, Capparaceae, Dipsacaceae, Geraniaceae, Heliotropiaceae, Juncaceae, Rosaceae, Santalaceae, Caprifoliaceae, Verbenaceae, Violaceae), due to inconsistency or unreliability for the Primorskaya lowland data, the family was detectable in our conditions. Thus, the families of Magnoliophyta that could potentially be found in our plots were identified.

### Supplementary text 2. MPD and statistical tests

Null distributions for MPD (the mean phylogenetic distance counted as the distance between two points along the evolutionary tree between all pairs of species from the set) obtained by permutations, estimating the significance of clustering as the fraction of permutations for MPD less or equal to the observed value. The significance of phylogenetic clustering was estimated by ranking MPD for the set of species under interest in the distribution of null values. To identify the null distribution for MPD, the same number of pairs were randomly subsampled from the species pool and represented in the phylogenetic tree 10,000 times. Second, combinatorial tests were also used (see the next section).

Comparison of means (salinity [electrical conductivity] and bare ground area) between microhighs and microlows at the later and earlier key sites was performed with the nonparametric Mann-Whitney U-test.

To identify Magnoliophyta families and orders that were significantly overrepresented or underrepresented in some species and families, respectively, the probability of picking the same or more/less species or families from this family or order were calculated from the entire pool of species and families, respectively. This was done by presuming that there are N species in a pool, and K of them are from the family under consideration, and there are n species in the set of interest with k of them from this family. The probability P_k_ that k species from this family arose in this set by chance was calculated by the formula (1) of hypergeometric probability:

$P(k)=\frac{C_{K}^{k}\times C_{N-K}^{n-k}}{C_{N}^{n}}$ (1)

Then, the probability Pover for k or more species from this family to arise in this set by chance was a sum of P(i), i $\in$[k, min(K, n)], and the probability Punder for k or more species from this family to arise in this set by chance was a sum of p(i), i $\in$[0, k]. If Pover (Punder) was low, it was unlikely that k or more/less species from this family appeared among K species of the set of interest by chance. Presumably, families with a Pover (Punder) ≤ 0.05 for a particular experimental 2×2 m plot were overrepresented (underrepresented) on this set.

### Supplementary list of references

1. Tejmurov, A. A. & Mirzaev, D. M. The systematic structure of the halophilic component of the flora of the Primorskaya lowland of Dagestan. *Izv. Dagest. Gos. Pedagog. Univ. Estestv. i tochnye Nauk.* **3**, 29–33 (2013).

2. Soltanmuradova, Z. I. & Teimurov, A. A. Taxonomic structure of the flora of the Primorskaya Lowland of the Republic of Dagestan. *South Russ. Ecol. Dev.* **3**, 38 (2010).

3. Lazareva, V. G. The structure and dynamics of the vegetation cover of the North-Western Caspian: problems of protection and rational use. (Astrakhan State Pedagogical University, 2000).

4. Bakhtasheva, N. M. *Conspectus of the flora of Kalmykia*. (Kalmyk State University, 2012).

5. Murtazaliev, R. *Conspectus of the Dagestan flora. Lycopodiaceae - Urticaceae*. **1**, (Epokha, 2019).

6. Murtazaliev, R. *Conspectus of the Dagestan flora. Euphorbiaceae - Dipsacaceae*. **2**, (Epokha, 2019).

7. Murtazaliev, R. *Conspectus of the Dagestan flora. Campanulaceae - Hippuridaceae*. **3**, (Epokha, 2019).

8. Murtazaliev, R. *Conspectus of the Dagestan flora. Melanthiaceae - Acoraceae*. **4**, (Epokha, 2019).

9. Mirzaev, D. M. Halophytes of the Primorsky Lowland (ecological-biological and geographical analysis). (Dagestan State University, 2013).

10. Murtazaliev, R. A. Chronicle of nature. Observations and research materials in the natural complexes of the Dagestansky reserve and the Agrakhansky, Samursky and Tlyaratinsky reserves in 2011. in *List of plant species of the site ‘Kizlyarsky Bay’ of the Daghestan State Nature Reserve* 109–116 (Federal State Budget Institution ‘Daghestan State Nature Reserve’, 2012).

11. Chase, M. W. *et al.* An update of the Angiosperm Phylogeny Group classification for the orders and families of flowering plants: APG IV. *Bot. J. Linn. Soc.* **181**, 1–20 (2016).

12. Anenkhonov, O. A. *et al.* *Keys to Plants of Buryatia*. (2001).

13. Anenkhonov, O. A. & A.Yu., K. C4 plants in steppe communities of Transbaikalia. in *Results and prospects of geobotanical studies in Siberia* (ed. Zibzeev, E. G.) 9–10 (Geo, 2019).

14. Grigore, M. N., Ivanescu, L. & Toma, C. *Halophytes: An integrative anatomical study*. *Halophytes: An Integrative Anatomical Study* (2014). doi:10.1007/978-3-319-05729-3

15. Ghaffari, S. M., Balaei, Z., Chatrenoor, T. & Akhani, H. Cytology of SW Asian Chenopodiaceae: new data from Iran and a review of previous records and correlations with life forms and C4 photosynthesis. *Plant Syst. Evol.* **301**, 501–521 (2015).

16. Lyubimov, V. Y. & Biel, K. Y. Metabolic strategy of annual desert plants: Adaptive phenomenon of CAM and C4 photosynthesis functioning in a leaf. in *Stress Responses in Plants: Mechanisms of Toxicity and Tolerance* 217–246 (2015). doi:10.1007/978-3-319-13368-3_9

17. Osborne, C. P. *et al.* A global database of C4 photosynthesis in grasses. *New Phytol.* **204**, 441–446 (2014).

18. Voznesenskaya, E. V. Ultrastructure of assimilating organs of some species of the family Chenopodiaceae II. *Bot. Zhurnal* **61**, 1546–1557 (1976).

19. Glagoleva, T. A. & Chulanovskaya, M. V. Photosynthetic metabolism of plants of the fam. Chenopodiaceae of arid saline areas. *Physiol. rasteniy* **39**, 672–679 (1992).

20. Melikyan, A. ., Avakyan, K. G. & Dildaryan, B. I. Problems of taxonomy and ecology of the family Frankeniaceae. *Biol. zhurnal Armen.* **XXX**, 46–57 (1977).

21. Akhani, H., Trimborn, P. & Ziegler, H. Photosynthetic pathways in Chenopodiaceae from Africa, Asia and Europe with their ecological, phytogeographical and taxonomical importance. *Plant Syst. Evol.* **206**, 187–221 (1997).

22. Akhani, H. & Ziegler, H. Photosynthetic pathways and habitats of grasses in Golestan National Park (NE Iran), with an emphasis on the C4-grass dominated rock communities. *Phytocoenologia* **32**, 455–501 (2002).

23. Akhani, H., Malekmohammadi, M., Mahdavi, P., Gharibiyan, A. & Chase, M. W. Phylogenetics of the Irano-Turanian taxa of Limonium (Plumbaginaceae) based on ITS nrDNA sequences and leaf anatomy provides evidence for species delimitation and relationships of lineages. *Bot. J. Linn. Soc.* **171**, 519–550 (2013).

24. Pyankov, V. I. & Vakhrusheva, D. V. Ways of primary fixation of CO2 in C4 plants of the Chenopodiaceae family in the arid zone of Central Asia. *Fiziol. rasteniy* **36**, 228–238 (1989).

25. Pyankov, V. I., Gunin, P. D., Tsoog, S. & Black, C. C. C4 plants in the vegetation of Mongolia: Their natural occurrence and geographical distribution in relation to climate. *Oecologia* **123**, 15–31 (2000).

26. Pyankov, V. I., Ziegler, H., Akhani, H., Deigele, C. & Lüttge, U. European plants with C4 photosynthesis: Geographical and taxonomic distribution and relations to climate parameters. *Bot. J. Linn. Soc.* **163**, 283–304 (2010).

27. Laktionov, A. P. *Flora of the Astrakhan region*. (Publishing House Astrakhan University, 2009).

28. Cherepanov, S. K. *Vascular plants of Russia and adjacent states (within the former USSR)*. (Cambridge University Press., 1995).

29. Govaerts, R., Nic Lughadha, E., Black, N., Turner, R. & Paton, A. The World Checklist of Vascular Plants, a continuously updated resource for exploring global plant diversity. *Sci. Data* **8**, 215 (2021).

30. POWO. Plants of the World Online. Facilitated by the Royal Botanic Gardens, Kew. *Board of Trustees of the Royal Botanic Gardens, Kew* (2022).

31. Rodin, L. E. *The vegetation of the deserts of Western Turkmenistan.* (Publishing house of the Academy of Sciences of the USSR, 1963).

32. Nechaeva, N. T., Vasilevskaya, V. K. & K.G., A. *Life forms of plants of the Karakum desert.* (Nauka, 1973).

33. Ivanov, V. V. *Keys to Plants of the Northern Caspian Region: Marevye, Liliaceae*. (Nauka, 1993).

34. Akzhigitova, N. I. *Halophilic vegetation of Central Asia and its indicative properties*. (FAN, 1982).

35. Magomedov, M. M. Cenosis-forming role of tree-like shrubs (Tamarix meyeri Boiss., T.ramosissima Ledeb.) in arid territories of the North-Western Caspian region. (2012).

36. Viktorov, S. V. & Remezova, G. L. *Indicator Geobotany*. (Lomonsov Moscow State University, 1988).

37. Baideman, I. N. & Preobrazhenskiy, A. S. Interdependence of the development of soils and vegetation in the Kura-Araks lowland. *Tr. Bot. instituta im. V.l. Komar. Akad. Nauk sssr. Seriya 3 Geobot.* **11**, 118–164 (1957).

38. Beideman, I. N., Bespalova, Z. G. & Rakhmanina, A. T. *Ecological and geobotanical sketch of the vegetation of the Mil steppe*. *Ecological-geobotanical and agromeliorative studies in the Kura-Araks lowland of the Transcaucasus (natural and anthropogenic changes in plant communities, water regime and plant root systems)* (AS of the USSR, 1962).
